# Comparison of child and family reports of health-related quality of life in pediatric acute lymphoblastic leukemia patients after induction therapy

**DOI:** 10.1186/s12887-020-02287-3

**Published:** 2020-08-19

**Authors:** Shohei Nakajima, Iori Sato, Takafumi Soejima, Katsuyoshi Koh, Motohiro Kato, Yasuhiro Okamoto, Toshihiko Imamura, Miho Maeda, Yasushi Ishida, Atsushi Manabe, Kiyoko Kamibeppu

**Affiliations:** 1 Department of Family Nursing, Division of Health Sciences and Nursing, Graduate School of Medicine, The University of Tokyo, 7-3-1 Hongo, Bunkyo-ku, Tokyo, 113-0033 Japan; 2 Department of Health Quality and Outcome Research, Global Nursing Research Center, Graduate School of Medicine, The University of Tokyo, 7-3-1 Hongo, Bunkyo-ku, Tokyo, 113-0033 Japan; 3 Department of Hematology/Oncology, Saitama Children’s Medical Center, 1-2 Shin-toshin, Chuo-ku, Saitama, 330-8777 Japan; 4 Children’s Cancer Center, National Center for Child Health and Development, 2-10-1 Okura, Setagaya-ku, Tokyo, 157-8535 Japan; 5 Department of Pediatrics, Kagoshima University Graduate School of Medical and Dental Sciences, 8-35-1 Sakuragaoka, Kagoshima, 890-8520 Japan; 6 Department of Pediatrics, Graduate School of Medical Science, Kyoto Prefectural University of Medicine, 465 Kajii-cho, Kawaramachi-Hirokoji, Kamigyo-ku, Kyoto, 602-8566 Japan; 7 Department of Pediatrics|, Nippon Medical School, 1-1-5 Sendagi, Bunkyo-ku, Tokyo, 113-8603 Japan; 8 Pediatric Medical Center, Ehime Prefectural Central Hospital, 83 Kasuga-machi, Matsuyama-shi, Ehime 790-0024 Japan; 9 Department of Pediatrics, Graduate School of Medicine, Hokkaido University, Kita 15, Nishi 7, Kita-ku, Sapporo, Hokkaido 060-8638 Japan

**Keywords:** Agreement, Dyadic analysis, Neoplasms, Patient reported outcome measures, Pediatric hospitals, Quality of life

## Abstract

**Background:**

This study aims at determining the health-related quality of life (HRQOL) of children with acute lymphoblastic leukemia (ALL) after the induction therapy, assessing the agreement between child self-reports and family proxy-reports HRQOL, and determining the factors related to this agreement, especially child age, family attendance, and children’s social relationships beyond the family.

**Methods:**

We analyzed questionnaire data (2012–2017) from the Japanese Pediatric Leukemia/Lymphoma Study Group’s clinical study (ALL-B12). Participants were children with B-cell precursor ALL aged 5–18 and their family members, who mostly took care of the child during hospitalization. Participants answered the Pediatric Quality of Life Inventory™ (PedsQL™) Generic Core Scales (PedsQL-G), and Cancer Module (PedsQL-C) to measure pediatric HRQOL. We calculated the differences between child self-reported and family proxy-reported subscale scores along with intraclass correlation coefficients (ICC). We conducted multiple regression analyses according to all participant pairs and age groups (young children, school age, and adolescents), with ICCs for all PedsQL-G subscales (ICC-G) and all PedsQL-C subscales (ICC-C) as the outcome variables.

**Results:**

Five hundred twenty-two pairs of children and their families were analyzed. We observed a moderate level of agreement on most PedsQL subscales between child self-reports and family proxy-reports; however, worry had the weakest agreement for all PedsQL subscales (ICC = .32, 95% confidence interval = .24–.40). The agreement of ICC-C was positively related to family attendance in the hospitalization, only for the young children group (*B* = .185, *p* = .003).

**Conclusions:**

We observed some differences between child self-reports and family proxy-reports of HRQOL of children with ALL. Both child self-reports and family proxy-reports captured HRQOL in the induction therapy. We suggest that attending to young children’s hospitalization affects the level of agreement between reports on their HRQOL.

## Background

Acute lymphoblastic leukemia (ALL) is the most common type of childhood cancer, accounting for 27.3% of pediatric cases [1]. Treatment for ALL, divided into the stages of induction therapy, intensive therapy, and maintenance therapy, is commonly recommended to be provided for at least 2 years from diagnosis, and some patients receive hematopoietic stem cell transplantation [2, 3]. The stratification of risk groups’ treatment and advancements in the use of multiagent and intrathecal chemotherapy, and so on, led to a dramatic increase in the five-year survival rate, which is now approximately 90% [2, 4, 5]. With the rise of the survival rate, researchers conducting clinical trials have increasingly valued determining time-to-event outcomes, such as overall and disease-free survival rate, as well as patients’ subjective outcomes, such as the quality of life, which directly affected to the extent that they experience to receive treatment and undergo painful procedures [6]. The American Society of Clinical Oncology defined quality of life in cancer patients as the second most important measure after overall and survival rate, and mentioned the importance of maintaining patients’ quality of life [7]. One particularly important factor is the health-related quality of life (HRQOL), a multidimensional construct broadly conceptualized as the effect of a disease and its treatment on individuals’ physical, emotional, and social well-being [8, 9]. The scales for assessing HRQOL are generally divided into two types. First are “generic scales,” focused on children’s general health condition regardless of the presence of a specific disease or disorder. Generic scales can be used to compare the HRQOL of children with a variety of diseases or none at all [10]. The second type are “disease-specific scales,” which focus on the domains of health affected by a specific illness and its treatment. Disease-specific scales are able to precisely pinpoint the effects of a given disease on children’s health and functioning [10, 11].

A recommended strategy to investigate HRQOL is through subjective assessment, however, it is important to understand the degree of agreement or mismatch between HRQOL as assessed by children (i.e., child self-reports) and family members (family proxy-reports), especially when considering the age, developmental stage, disease condition, cognitive ability, and linguistic skill of children [12–14]. The degrees of agreement or mismatch between child self-reports and family proxy-reports have been found to vary according to family, child characteristics, and physical and psychosocial domains [15], suggesting the importance of clarifying the level of agreement between the reports of HRQOL among children with ALL.

One previous study examined the level of agreement between child self-reported and family proxy-reported HRQOL among pediatric patients with ALL, and determined that children’s age, gender, treatment intensity, and phase of the treatment were related to this agreement [16]. Given the effect of children’s age, it is possible that the agreement between reports depends on the time family members spend with their children, as it is likely that spending more time with the children leads to a better understanding of their HRQOL [15, 16]. Therefore, we can suppose that the amount of time children spend with their family members will enhance the agreement between their reports. Additionally, we assume that the amount of time that children spend in social relationships outside the family, such as in nursery or elementary schools, affects the agreement between reports—that is, the less time children spend with their family and the more children interaction is focused on friends rather than family, the weaker the degree of agreement between reports [15].

To investigate issues related to the agreement between reports, we drew on the clinical trial data of the Japanese Pediatric Leukemia/Lymphoma Study Group’s “ALL-B12: A Multi-Center Phase II/III Study in Children with Newly Diagnosed B-cell Precursor ALL” (hereinafter, ALL-B12). In this trial, HRQOL was set as the secondary outcome, which was a novelty for a large clinical trial in the Japanese Pediatric Leukemia/Lymphoma Study Group. The ALL-B12 is expected to elucidate the randomized treatments effect of HRQOL. However, to clarify the agreement between child self-reports and family proxy-reports of HRQOL, and the factors related to this agreement, we used the ALL-B12 at baseline before randomization of therapies, that is, post-induction therapy.

The induction therapy comprises multidrug chemotherapy and invasive interventions, such as repeated lumbar punctures and bone-marrow punctures [17]. These are known for potential serious side effects, such as pyrexia, nausea, and diarrhea [18]. Furthermore, patients undergoing the induction therapy spend long periods in the hospital and have notable limitations in daily life [19]. Given these conditions, we hypothesized that HRQOL would reach its lowest level during the induction therapy in comparison to other phases of the treatment. Accordingly, longitudinal HRQOL surveys with children with ALL have shown that, HRQOL was worst at one-month post-diagnosis (Children’s Oncology Group, COG) [20] or 35 days post-diagnosis (Japan Association of Childhood Leukemia Study, JACLS) [21] before the end of the induction therapy, compared with consolidation therapy, maintenance therapy, or after therapy. Most studies on the HRQOL of children with ALL have almost exclusively focused on the post-chemotherapy period [22]; however, in the last decade, more studies started to focus on the induction therapy ([20]; van [23]). Thus, we sought to clarify the factors involved in the agreement between child reports and family proxy-reports of HRQOL at the induction therapy.

Therefore, the purposes of this study were (1) to clarify the agreement between child self-reports and family proxy-reports of HRQOL among children with ALL who had received the induction therapy, and (2) to determine the factors related to this agreement. Given prior findings, we were particularly interested in the influence of children’s age, family attendance, and social relationships outside of the family. Understanding the extent of the agreement between child self-reports and family proxy-reports of HRQOL at the induction therapy and the factors related to it, we can interpret HRQOL after further treatment of the induction therapy.

## Methods

### Data

We obtained and analyzed the data from questionnaires answered by children and their families in the ALL-B12. The induction therapy of ALL-B12 comprises approximately a month of combined chemotherapy, including steroid pre-phase and the induction multidrug therapy [3]. The following inclusion criteria were used to select relevant child–family pairs: (1) children with B-cell Precursor ALL and their family had entered ALL-B12 from December 2012 to November 2017; (2) children with B-cell Precursor ALL were aged 5–18 years; and (3) both children and their families returned all and completed questionnaires to the Center for Quality of Life Research after the induction therapy (about 6 weeks from the start of the therapy).

Children and their families were given questionnaires and a self-addressed stamped envelope by their attending physicians before and after 2 weeks of the scheduled date of the end of the induction therapy. Children and their families answered the questionnaires within 4 weeks from receiving them, and then sent them to the Research Center using the self-addressed stamped envelope.

### Measurements

#### HRQOL

We used the Pediatric Quality of Life Inventory Japanese Version (PedsQL) [24, 25] to measure pediatric HRQOL. PedsQL was developed using surveys with many children, including healthy children and children with various types of disease (e.g., cancer), their families, and healthcare professionals. It was designed to measure pediatric HRQOL in the past month [9, 10, 26]. PedsQL Generic Core Scales (PedsQL-G) [24] measure general HRQOL using the World Health Organization’s (WHO) definition of health—“Health is a state of complete physical, mental and social well-being and not merely the absence of disease or infirmity”—with the added dimensions of role/school functioning [27]. It comprises 21 items for children aged between 5 and 7 and 23 items for those between 8 and 18 in four functioning subscales (i.e., *physical, emotional, social,* and *school functioning*). We also used the PedsQL Cancer Module (PedsQL-C) [25], which focuses on the dimensions of health affected by pediatric cancer and its treatment. It comprises 26 items for those aged from 5 to 12 and 27 items for those from 13 to 18 in eight subscales (i.e., level of *pain, nausea, procedural anxiety, treatment anxiety, worry, cognitive problems, perceived physical appearance,* and *communication*). Children and families answered each item on a 5-point Likert scale, where *0 = no problem, 1 = almost never, 2 = sometimes, 3 = often, and 4 = almost always*. However, children aged from 5 to 7 answered each item on a 3-point Likert scale adopting faces corresponding to frequencies: *a smiley face* representing “*0 = no problem*,” *a neutral face* representing “*2 = sometimes*,” and *a frowning face* representing “*4 = almost always*.” The children answered the questionnaires with the help of their families or medical staff, if necessary. In such cases, we requested family members to first answer their questionnaires before helping children. Based on the PedsQL scoring algorithm [28], we calculated the average score for each item in the subscales of both the child self-reports and family proxy-reports, and then transformed them to a 0–100 scale, where high scores indicated high HRQOL. If participants answered fewer than 50% of items in a subscale, the subscale was considered to have missing scores. For all domains, Cronbach’s alphas exceeded .80 for both children and family members. These scales were, therefore, adequately valid in target participants.

#### Family attendance ratio during children’s hospitalization

Family members, more precisely, the person who mostly took care of the child during hospitalization, reported the number of days of hospitalization and their own attendance during this period in the previous month. From this information, we calculated the family attendance ratio by dividing the number of days of family attendance in the previous month by the number of days of hospitalization in that month.

#### Social relationships and nursery/school characteristics

The children reported the number of friends they thought to have (*1 = none, 2 = one friend, 3 = 2–3 friends, 4 = over 4 friends*), whereas their family answered whether children were attending a nursery or school and how present they were *(1 = almost always absent, 2 = present one-third of the time, 3 = present two-thirds of the time, 4 = almost always present*).

#### Demographic variables

The children reported their age and gender, whereas family members answered their age and relationship to the child.

### Statistical analyses

We used R ver. 3.5.0 [29] and set the significance level to 5% (two-tailed test). As a first step in the analysis, we calculated descriptive statistics classifying all children by their age group, as follows: young children (aged between 5 and 7), school-age children (aged between 8 and 12), and adolescents (aged between 13 and 18).

To verify the agreement between the child self-reports and family proxy-reports, we calculated the differences in each PedsQL subscale between the types of reports using *t*-statistics and their 95% confidence intervals (95% CI). We also calculated intraclass correlation coefficients (ICCs) between child self-reports and family proxy-reports, and their 95% CIs based on a two-way random effects model [30]. Using Landis et al.’s criteria, ICCs were categorized as weak (≤ .40), moderate (.41–.60), substantial (.61–.80), and almost perfect (≥ .81) [31].

To explore the factors influencing the agreement between child self-reports and family proxy-reports for each family, we conducted multiple regression analyses for all participant pairs and age groups. The explanatory variables were the seven items previously mentioned, including (1) child’s age, (2) child’s gender, (3) family member’s age, (4) family member’s relationship to the child, (5) number of friends, (6) attendance at nursery/school, and (7) family attendance ratio; all were simultaneously entered into the regression analyses.

For outcome variables, we used several indicators of agreement, according to previous studies, including the absolute difference between PedsQL scores and the ICCs for all subscales between child self-reports and family proxy-reports. Previous studies have used both the difference [16], and absolute difference [32] in scores as indicators of agreement. In this study, the raw difference between reports was deemed inappropriate for multiple regression because larger numerical values would indicate less agreement when the difference was positive, but greater agreement when the difference was negative. Thus, we used instead the absolute values of the difference for the subscales of the PedsQL. Finally, we calculated the ICCs for child self-reports and family proxy-reports of all 4 PedsQL-G subscales (ICC-G), which was defined as the agreement for overall pediatric HRQOL. Similarly, we calculated the ICCs of child self-reports and family proxy-reports of all 8 PedsQL-C subscales (ICC-C), which we defined as the agreement of pediatric HRQOL specific to cancer and its treatment.

### Ethical considerations

The ALL-B12 was approved by the institutional review board of the Japanese Society of Pediatric Hematology/Oncology and the Graduate School of Medicine, University of Tokyo. It also received approval from each participating medical center and hospital. We obtained permission to use the anonymized data of participants from the Japanese Society of Pediatric Hematology/Oncology ALL committee. We made sure to protect participants’ personal information by conducting all de-identified data handling and analyses in the Research Center.

## Results

### Participants (Table 1)

A total of 545 pairs of children and family members returned questionnaires, twenty-three pairs were excluded for not fully completing the PedsQL items, either for the child self-report (*n* = 17) or for the family proxy-report (*n* = 6). Thus, a total of 522 pairs were used in the analysis. The mean and standard distribution days of the differences between child self-reports and family proxy-reports in the time taken to complete the questionnaires were − 0.22, and 2.76, respectively.

**Table 1:** Demographic of children and their family members (*N* = 522)

	All Children (***N*** = 522) *n* (%) or *mean* ± *SD*		Young child (***n*** = 205) *n* (%) or *mean* ± *SD*		School age (***n*** = 215) *n* (%) or *mean* ± *SD*		Adolescent (***n*** = 102) *n* (%) or *mean* ± *SD*	
Child’s age (years)	9.2	± 3.5	5.9	± 0.9	9.7	± 1.5	14.8	± 1.4
Child’s sex								
Male	266	(51.0)	109	(53.2)	107	(49.8)	50	(49.0)
Female	256	(49.0)	96	(46.8)	108	(50.2)	52	(51.2)
Family member’s age	40.1	± 6.4	37.3	± 4.9	40.5	± 6.3	44.9	± 6.3
Family member’s relationship to the child								
Mother	481	(92.1)	190	(92.7)	197	(91.6)	94	(92.1)
Father	35	(6.7)	13	(6.3)	15	(7.0)	7	(6.9)
Other	6	(1.2)	2	(1.0)	3	(1.4)	1	(1.0)
Number of friends (child perspective)								
None	31	(6.3)	10	(5.1)	15	(7.4)	6	(6.2)
One friend	23	(4.6)	10	(5.1)	10	(5.0)	3	(3.1)
2—3 friends	134	(27.1)	72	(36.5)	45	(22.4)	17	(17.5)
over 4 friends	307	(62.0)	105	(53.3)	131	(60.2)	71	(73.2)
Attending a nursery or school								
Yes	426	(85.5)	143	(70.1)	200	(98.0)	83	(92.2)
No	72	(14.5)	61	(29.9)	4	(2.0)	7	(7.8)
Presence at nursery or school								
Almost always presence	132	(31.3)	43	(29.5)	73	(37.8)	16	(19.3)
Presence two-thirds of the time	87	(20.6)	21	(14.4)	48	(24.9)	18	(21.7)
Presence one-third of the time	50	(11.8)	12	(8.2)	33	(17.1)	5	(6.0)
Almost always absence	153	(36.3)	70	(47.9)	39	(20.2)	44	(53.0)
Number of days of hospitalization in the previous month (days)	29.4	± 3.9	29.4	± 3.5	29.1	± 4.7	29.8	± 2.8
Number of days of family attendance in the previous month (days)	18.7	± 13.8	23.0	± 12.1	18.8	± 13.9	10.0	± 12.8
Family attendance ratio during children’s hospitalization	0.64	± 0.45	0.78	± 0.39	0.65	± 0.45	0.34	± 0.43

Notes: Excluding missing values.

Abbreviations: *SD* Standard deviation

Children’s mean age was 9.2 years, whereas that of the family members was 40.1 years [range: 16–68]. Two hundred sixty-six children were male (51%). Regarding family relationship, the vast majority were mothers (481; 92.1%), 35 were fathers (9.7%), and the rest were grandparents or siblings. By age group, 205 were young children (39.3%), 215 were school-aged children (41.2%), and 102 were adolescents (19.5%). Children who had received 6 weeks of the induction therapy tended to be hospitalized for most of the past month. The mean number of days of family attendance in the past month was 18.7 days, and 289 family members (55.4%) spent every day with their children while they were in the hospital (i.e., 100% family attendance ratio). By age group, the family attendance ratio was smaller for adolescents than for school-aged children and young children.

### Score distributions and agreements between child self-reports and family proxy-reports

Physical functioning was the lowest score for all PedsQL subscales reported children (mean = 54.4) and family members (mean = 45.0). To obtain the differences, we subtracted the family proxy-reported scores from the child self-reported scores for all PedsQL subscales. Child self-reported role/school functioning scores (mean = 63.8) were lower than family proxy-reported scores (mean = 78.1) on all PedsQL-G subscales within the young children group. Also, child self-reported procedural anxiety scores (mean = 63.9) was significantly lower than that of the family proxy-reports (mean = 69.5) in PedsQL-C subscales. We observed significant differences in physical functioning (differences = 9.30; 95% CI: 7.4–11.2), emotional functioning (differences = 5.40; 95% CI: 3.2–7.6), social functioning (differences = 2.69; 95% CI: 0.8–4.6), pain and hurt (differences = 3.48; 95% CI: 1.1–5.9), nausea (differences = − 3.65; 95% CI: − 5.5–-1.8), procedural anxiety (differences = − 5.58; 95% CI: − 7.8–-3.4), and cognitive problems (differences = − 1.27; 95% CI: − 5.8–-1.9) among all children (Table 2).

**Table 2:** Agreement of child self-reports and family proxy-reports of pediatric health-related quality of life (*N* = 522)

		*n*	Child self-reports		Family proxy-reports		Diffences^a)^	*t-*value^b)^	95% CI for Differences (*lower*) (*upper*)		ICC^C)^	95% CI for ICC (*lower*) (*upper*)	
PedsQL-G (Generic Core Scales)													
Physical functioning	All Children	518	**54.4**	**± 25.5**	45.0	± 26.2	9.3	9.64	7.4	11.2	.60	.47	.70
	Young Child	204	**56.9**	**± 24.5**	47.4	± 27.3	9.3	6.08	6.3	12.3	.61	.45	.72
	School age	212	**53.0**	**± 26.9**	44.7	± 26.4	8.2	5.18	5.1	11.3	.60	.47	.70
	Adolescent	102	**52.7**	**± 24.1**	41.1	± 23.1	11.6	5.88	7.7	15.6	.58	.30	.73
Emotional functioning	All Children	518	**68.6**	**± 26.1**	63.2	± 24.9	5.4	4.84	3.2	7.6	.49	.42	.56
	Young Child	204	64.3	± 27.2	65.8	± 25.4	−1.3	−0.71	−5.1	2.4	.48	.36	.58
	School age	212	**72.2**	**± 24.9**	62.1	± 25.7	10.0	5.98	6.7	13.3	.50	.35	.62
	Adolescent	102	**69.9**	**± 25.1**	60.5	± 21.6	9.4	4.39	5.1	13.6	.54	.34	.68
Social functioning	All Children	494	**84.5**	**± 18.3**	82.0	± 21.4	2.7	2.76	0.8	4.6	.41	.33	.48
	Young Child	193	81.8	± 18.2	82.7	± 21.0	−0.8	−0.51	−3.9	2.3	.40	.28	.51
	School age	209	**86.8**	**± 17.9**	80.6	± 22.4	6.3	4.37	3.4	9.1	.46	.34	.57
	Adolescent	92	85.4	± 18.5	83.8	± 20.2	1.9	0.80	−2.8	6.6	.32	.12	.49
School functioning	All Children	391	75.1	± 27.2	74.5	± 26.0	1.3	1.02	−1.2	3.7	.55	.48	.61
	Young Child	119	63.8	± 31.0	**78.1**	**± 25.4**	−12.2	−5.45	− 16.7	−7.8	.56	.35	.71
	School age	196	**82.7**	**± 21.9**	75.0	± 25.7	7.2	4.61	4.1	10.3	.54	.41	.65
	Adolescent	76	**74.4**	**± 26.6**	66.6	± 21.6	7.2	2.60	1.7	12.7	.57	.40	.71
PedsQL-C (Cancer Module)													
Pain and hurt	All Children	521	**74.6**	**± 31.3**	71.1	± 29.9	3.5	2.87	1.1	5.9	.59	.53	.64
	Young Child	204	72.8	± 32.8	74.7	± 28.5	−1.9	−0.97	−5.8	2.0	.59	.49	.67
	School age	215	**77.7**	**± 30.2**	70.4	± 31.2	7.3	3.79	3.5	11.1	.56	.45	.65
	Adolescent	102	**71.5**	**± 30.0**	65.3	± 28.8	6.1	2.62	1.5	10.8	.67	.54	.76
Nausea	All Children	520	65.6	± 28.3	**69.4**	**± 28.3**	−3.7	−3.80	−5.5	−1.8	.70	.65	.74
	Young Child	203	61.8	± 26.9	**76.0**	**± 24.7**	−14.0	−10.04	− 16.8	− 11.3	.61	.28	.78
	School age	215	**71.0**	**± 28.6**	67.3	± 30.4	3.6	2.68	1.0	6.3	.77	.71	.82
	Adolescent	102	62.0	± 29.1	60.4	± 27.8	1.6	0.73	−2.7	5.9	.70	.59	.79
Procedural anxiety	All Children	520	63.9	± 32.2	**69.5**	**± 30.0**	−5.6	−4.99	−7.8	−3.4	.65	.59	.71
	Young Child	205	50.9	± 30.7	**64.1**	**± 30.9**	−13.2	−7.45	−16.7	−9.7	.61	.40	.73
	School age	214	69.5	± 32.3	70.2	± 30.4	−0.6	−0.37	− 4.0	2.7	.69	.61	.76
	Adolescent	101	78.4	± 24.4	78.9	± 24.3	− 0.6	−0.25	−5.2	4.1	.53	.38	.66
Treatment anxiety	All Children	509	86.3	± 22.5	85.6	± 21.3	0.6	0.65	−1.2	2.3	.58	.52	.64
	Young Child	199	82.6	± 25.7	84.3	± 22.4	−2.1	−1.39	−5.0	0.9	.62	.53	.70
	School age	212	87.6	± 21.3	86.3	± 20.6	1.3	0.98	−1.3	3.9	.58	.48	.66
	Adolescent	98	**91.0**	**± 16.4**	86.8	± 20.5	4.3	2.26	0.5	8.1	.48	.31	.61
Worry	All Children	515	65.6	± 30.4	66.3	± 31.7	−0.5	−0.33	−3.7	2.6	.32	.24	.40
	Young Child	200	64.8	± 32.6	**75.7**	**± 29.7**	−11.1	−4.12	−16.4	−5.8	.24	.10	.36
	School age	214	**69.4**	**± 28.0**	64.6	± 31.3	5.0	2.14	0.4	9.5	.34	.22	.45
	Adolescent	101	**59.2**	**± 30.0**	51.0	± 30.3	8.7	2.80	2.5	15.0	.44	.27	.58
Cognitive problems	All Children	508	75.6	± 23.7	**79.4**	**±22.5**	−3.9	−3.92	−5.8	−1.9	.53	.46	.59
	Young Child	199	74.1	± 28.0	**83.6**	**± 19.7**	−9.6	−6.04	−12.7	−6.5	.43	.26	.56
	School age	212	78.1	± 26.3	78.5	± 22.2	−0.5	−0.34	−3.1	2.2	.64	.55	.71
	Adolescent	96	73.3	± 30.9	72.4	± 24.7	0.5	0.20	−4.5	5.5	.50	.33	.63
Perceived physical appearance	All Children	488	73.1	± 27.9	74.2	± 27.6	−1.3	−0.98	−3.8	1.3	.48	.40	.54
	Young Child	203	71.1	± 28.0	**81.7**	**± 23.1**	−10.6	−5.70	− 14.3	−7.0	.43	.28	.55
	School age	197	**75.8**	**± 26.3**	70.1	± 29.6	6.0	3.28	2.4	9.7	.57	.46	.66
	Adolescent	88	71.6	± 30.9	66.6	± 28.4	4.0	1.13	−3.0	10.9	.41	.22	.57
Communication	All Children	483	63.4	± 30.7	63.6	± 29.0	−1.0	−0.78	−3.4	1.5	.58	.52	.64
	Young Child	197	55.8	± 31.1	**63.9**	**± 29.2**	−7.8	−4.08	−11.5	−4.0	.58	.48	.68
	School age	198	**65.8**	**± 29.2**	60.7	± 30.3	4.6	2.49	1.0	8.3	.61	.51	.69
	Adolescent	88	73.8	± 29.6	69.7	± 24.5	1.7	0.55	−4.3	7.7	.48	.30	.62

Notes: Excluding missing values. **Bold** scores indicated significantly higher than another reports.

^a^Mean of the differences subtracting family proxy-reports from child self-reports, ^b^Comparing children self-reports and family proxy-reports using paired *t*-test

Abbreviations: *CI* Confidence interval, *ICC* Intraclass correlation coefficient, *PedsQL* Pediatric Quality of Life Inventory™, *SD* Standard deviation

According to the ICCs, worry had the weakest agreement for all PedsQL subscales (ICC = .32). For the other subscales, the degree of agreement was moderate to substantial (ICC = .41–.70). By age group, social functioning among adolescents (ICC = .32) and worry among young children (ICC = .24) and school-aged children (ICC = .34) indicated weak agreement. The other PedsQL subscales indicated moderate to strong agreement. The mean ICC-G and ICC-C between child self-reports and family proxy-reports were 0.54 and 0.48, respectively.

### Factors related to the agreement between child self-reports and family proxy-reports

When we set the absolute difference between child self-reports and family proxy-reports as the outcome variable in the regression analysis, we observed no significant relationships and the coefficients of determination were nearly zero. The same finding was obtained when the ICC-G was used as the outcome variable (Table 3). However, when the ICC-C was set as the outcome, we found that the family attendance ratio (*B* = .185, *P*-value = .003) was significantly related to the agreement in the young children group (Table 4); there were no significant relationships in the school-aged and adolescent groups.

**Table 3:** Multiple regression analysis of predicting ICC-G (intraclass correlation coefficient of all 4 PedsQL Generic Core Scales subscales)

	All Children (***N*** = 522)			Young child (***n*** = 205)			School age (***n*** = 215)			Adolescent (***n*** = 102)		
	*B*	*p*	*VIF*	*B*	*p*	*VIF*	*B*	*p*	*VIF*	*B*	*p*	*VIF*
Child’s age	.006	.311	1.384	−.044	.285	1.449	.006	.732	1.132	−.022	.511	1.255
Child’s sex	.016	.645	1.034	.021	.730	1.090	−.006	.899	1.048	.100	.249	1.125
Family member’s age	−.002	.639	1.267	.002	.773	1.013	−.005	.193	1.095	.004	.661	1.179
Family member’s relationship to the child	−.068	.328	1.031	−.022	.870	1.076	−.011	.904	1.042	−.290	.136	1.146
Number of friends	−.008	.680	1.022	−.010	.802	1.042	−.025	.340	1.032	.027	.569	1.075
Attendance at nursery/school	−.017	.202	1.037	−.006	.829	1.466	−.028	.186	1.093	−.035	.333	1.208
Family attendance ratio	−.069	.080	1.144	−.046	.537	1.064	−.111	.043	1.069	−.019	.845	1.066
*R*^2^	.022	.319		.018	.948		.046	.356		.068	.665	
Adjust *R*^2^	.003			−.039			.005			−.028		

Notes: Excluding missing values.

Child’s sex: dummy code as 1 = Male, 0 = Female; Family member’s relationship to the child: dummy code as 1 = Mothers, 0 = Others; Number of friends: 1 = none, 2 = one friend, 3 = 2–3 friends, 4 = over 4 friends; Attendance at nursery/school: dummy code as 1 = Yes, 0 = No; Family attendance ratio was calculated that number of days of child hospitalization divided by number of days of family attendance in the previous a month.

Abbreviations: *B* Non-standardized partial regression coefficient, *PedsQL* Pediatric Quality of Life Inventory™, *R* Coefficient of determination, *VIF* Variance inflation factor

**Table 4:** Multiple regression analysis of predicting ICC-C (intraclass correlation coefficient of all 8 PedsQL Cancer Module subscales)

	All Children (***N*** = 522)			Young child (***n*** = 205)			School age (***n*** = 215)			Adolescent (***n*** = 102)		
	*B*	*p*	*VIF*	*B*	*p*	*VIF*	*B*	*p*	*VIF*	*B*	*p*	*VIF*
Child’s age	.002	.740	1.385	−.041	.221	1.449	−.017	.329	1.133	−.023	.418	1.255
Child’s sex	.010	.753	1.035	.013	.795	1.090	.024	.623	1.052	−.019	.797	1.125
Family member’s age	.004	.200	1.268	.009	.100	1.013	.001	.842	1.097	.010	.190	1.179
Family member’s relationship to the child	.041	.524	1.032	−.119	.286	1.076	.125	.176	1.042	.075	.656	1.146
Number of friends	−.021	.253	1.022	−.055	.081	1.042	.013	.625	1.032	−.059	.145	1.075
Attendance at nursery/school	−.018	.157	1.038	−.019	.384	1.466	−.018	.399	1.095	−.014	.656	1.208
Family attendance ratio	.020	.593	1.145	.185	.003	1.064	−.050	.359	1.072	−.091	.282	1.066
*R*^2^	.019	.438		.158	.003		.022	.818		.085	.506	
Adjust *R*^2^	.000			.109			−.020			−.009		

Notes: Excluding missing values.

Child’s sex: dummy code as 1 = Male, 0 = Female; Family member’s relationship to the child: dummy code as 1 = Mothers, 0 = Others; Number of friends: 1 = none, 2 = one friend, 3 = 2–3 friends, 4 = over 4 friends; Attendance at nursery/school: dummy code as 1 = Yes, 0 = No; Family attendance ratio was calculated that number of days of child hospitalization divided by number of days of family attendance in the previous a month.

Abbreviations: *B* Non-standardized partial regression coefficient, *PedsQL* Pediatric Quality of Life Inventory™, *R* Coefficient of determination, *VIF* Variance inflation factor

## Discussion

We investigated the agreements and the factors affecting it in child self-reports and family proxy-reports of HRQOL among children with ALL, especially young children, receiving the induction therapy.

### Participant demographics

We selected children and adolescents aged between 5 and 18, as the incidence of ALL has been found to be the second highest among 5–9-year-old children, after 1–4-year-old children, and decreases with age [33]. Furthermore, the gender ratio in terms of incidence has been reported as 1:1.3 (girl: boy) [4]. In line with these past findings, the largest group of participants was composed of young children, followed by school-aged and adolescents, and the number of boys was larger than that of girls. The ALL-B12 was conducted in almost all hospitals and centers treating childhood cancer in Japan. Therefore, we might safely conclude that there were no selection biases among participants—they may be considered suitably representative of children and adolescents aged from 5 to 18, having B-precursor ALL, and receiving the induction therapy.

### Agreement and discrepancy between child self-reports and family proxy-reports

Physical functioning had the lowest score of all PedsQL subscales, which is consistent with previous findings [13, 34]. This is likely due to the effects of the induction therapy. Furthermore, the family proxy-reports of physical functioning were significantly lower than child self-reports; this result shows that family members assessed their children’s physical functioning lower than the children themselves [16]. However, interestingly, the ICC of physical functioning was stronger than that of the other PedsQL-G subscales. The greater degree of concordance is perhaps due to physical functioning being more observable than more subjective dimensions of functioning, such as emotional and social functioning [6, 35]. More specifically, when children’s health status was poor, their family members tended to observe them more carefully [6]. Thus, although there were some differences between child self-reports and family proxy-reports, these differences were rather small, and the agreement was overall quite strong. Based on the results, we might say that family proxy-reports of children’s HRQOL are somewhat worse than child self-reports in terms of physical functioning, although this deserves more careful investigation.

Child self-reported role/school functioning scores were lower than family proxy-reported scores on all PedsQL-G subscales within the young children group. This may be because family members did not consider children’s absence from their nursery or school as much of a problem, as they were primarily concerned with their children’s disease and treatment, as mentioned in a previous qualitative study [36]. In contrast, children felt worse about being absent from their nursery or school compared to staying at the hospital for treatment.

We found that the child self-reported procedural anxiety scores were significantly lower than that of the family proxy-reports —this difference was the largest of all PedsQL subscales. This subscale concerned children’s feelings of fear and pain in having their blood sampled or having needles inserted in them. A previous Canadian study conducted among 260 children with ALL receiving treatment [22] similarly found that child self-reported procedural anxiety had lower scores than all other PedsQL-C subscales. The developmental literature of the Japanese version of the PedsQL-C [25] also found that child self-reported procedural anxiety was the lowest scoring domain of HRQOL among young children. Participants’ involvement in the induction therapy might be a justifiable reason for the lower scores, given that the induction therapy includes highly invasive examinations and combination treatments, such as repeated blood sampling, intrathecal chemotherapy [37], and bone-marrow aspiration [38].

Our present data showed that the agreement between child self-reports and family proxy-reports ranged from moderate to strong for almost all subscales. However, worry showed the weakest level of agreement in all age groups, especially within young children. Child self-reported worry score was lower than the family proxy-reported score. A possible reason is that family members might have felt that children would find it difficult to predict the side effects of the induction therapy or the likelihood of relapse; in contrast, children may have found it difficult to understand the future effects of the induction therapy, thus leading to a lower agreement.

A previous study including 51 families suggested that both discrepancy and agreement are important indices to consider when examining the consensus between child self-reports and family proxy-reports [39]. Indeed, we found differences between child self-reports and family proxy-reports in some domains of HRQOL among young children with ALL. This suggests that both reports are important for interpreting children’s HRQOL, as they allow us to draw an accurate portrait of HRQOL.

### Exploring the factors related to the agreement between child self-reports and family proxy-reports

The standard deviations of ICC-G and ICC-C between child self-reports and family proxy-reports were variable. Additionally, we found that, in the young children group, the agreement according to the ICC-C was stronger when the family attendance ratio was higher. We confirmed Eiser et al.’s proposal [15] that parents spend more time with younger children and, therefore, are expected to have a more comprehensive overview of their children’s physical, emotional, and social functioning, thus leading to greater agreement between parents and younger children. An alternative explanation for the effect of the family attendance ratio on the agreement between the reports in the young children group is that family members might have asked medical staffs (e.g., doctors, nurses) about their children’s symptoms while family members left their children’s side. However, doctor–child agreement has been found to be weaker than parent–child agreement [40], indicating that family members might have difficulty inferring their children’s HRQOL from objective symptoms reported by the medical staff. Therefore, the effect of the time spend with their children leads to a strong agreement between the reports based on family members direct observation of children’s symptoms.

Notably, no factors were related to agreement among all children or among school-aged children and adolescents. This is probably because school-aged children and adolescents do not spend so much time with their family, and their social interactions are predominantly outside of the family. Furthermore, children are likely to become increasingly separated from their family as they grow up. However, family members would ask school-aged children and adolescents about their symptoms, which is perhaps why the time they spend with children (e.g., family attendance) and social relationships outside family are not related with the agreement.

This study has some limitations. First, family attendance might not reflect the actual time spend by the family with the children because the questionnaire only assessed the number of “days” of family attendance. It would be necessary to add “hours and/or weekdays/weekends” to the family attendance ratio in a future study to improve our understanding of the effect of this factor on the agreement between reports. Second, approximately 40% of the children and their family members showed attrition in this study. Moreover, there were great discrepancy for filling of the questionnaire between reports. Our research couldn’t obtain the details of non-respondent’s data, because of the third-party institution on this clinical trial. A reason for this attrition might be children’s medical condition or higher psychological distress, which caused difficulty in answering the questionnaire on time. Further research should consider how to better retain these cases.

This study was a national survey conducted in numerous centers and hospitals treating pediatric cancer patients in Japan, and this study was the first to clarify the level of agreement between child self-reported and family proxy-reported HRQOL, and how this is influenced by the family attendance ratio during the induction therapy. In the future, it is important to discuss the HRQOL of children with ALL who are receiving the induction therapy while considering the results of this study.

## Conclusions

This study explored the agreement between child self-reported and family proxy-reported HRQOL in children with ALL at the induction therapy. Overall, we found a moderate level of agreement for most HRQOL subscales, despite some notable absolute differences. Furthermore, we found that a higher family attendance ratio was positively related to the agreement of cancer-specific HRQOL among young children. Both child self-reports and family proxy-reports could capture the actual HRQOL in the induction therapy. We suggest considering allowing for the presence of family members during hospitalization to interpret the agreements of HRQOL between child self-reports and family proxy-reports, especially when the patients are young.

## Data Availability

The dataset in this study is accessible at the corresponding author upon a reasonable request.

## Abbreviations

ALL: Acute lymphoblastic leukemia
CI: Confidence intervals
ICC: Intraclass correlation coefficient
HRQOL: Health-related quality of life
PedsQL: Pediatric Quality of Life Inventory™
